# Engineering *Vibrio alginolyticus* as a novel chassis for PHB production from starch

**DOI:** 10.3389/fbioe.2023.1130368

**Published:** 2023-02-07

**Authors:** Hong-Fei Li, Linyue Tian, Guoli Lian, Li-Hai Fan, Zheng-Jun Li

**Affiliations:** ^1^ College of Chemical Engineering, Fujian Engineering Research Center of Advanced Manufacturing Technology for Fine Chemicals, Fuzhou University, Fuzhou, China; ^2^ College of Life Science and Technology, Beijing University of Chemical Technology, Beijing, China; ^3^ Qingyuan Innovation Laboratory, Quanzhou, China

**Keywords:** amylase, metabolic engineering, poly-3-hydroxybutyrate, starch, *Vibrio alginolyticus*

## Abstract

*Vibrio alginolyticus* LHF01 was engineered to efficiently produce poly-3-hydroxybutyrate (PHB) from starch in this study. Firstly, the ability of *Vibrio alginolyticus* LHF01 to directly accumulate PHB using soluble starch as the carbon source was explored, and the highest PHB titer of 2.06 g/L was obtained in 18 h shake flask cultivation. Then, with the analysis of genomic information of *V. alginolyticus* LHF01, the PHB synthesis operon and amylase genes were identified. Subsequently, the effects of overexpressing PHB synthesis operon and amylase on PHB production were studied. Especially, with the co-expression of PHB synthesis operon and amylase, the starch consumption rate was improved and the PHB titer was more than doubled. The addition of 20 g/L insoluble corn starch could be exhausted in 6-7 h cultivation, and the PHB titer was 4.32 g/L. To the best of our knowledge, *V. alginolyticus* was firstly engineered to produce PHB with the direct utilization of starch, and this stain can be considered as a novel host to produce PHB using starch as the raw material.

## 1 Introduction

With the development of modern biotechnology and synthetic biology, microorganisms have been considered as important chassis cells for the production of biological products. While rational metabolic engineering of microorganisms greatly reduces the cost of production, the time required for cell growth and product synthesis during fermentation process is likely to be a potential limitation (Hoff et al., 2020). As is all known, *Escherichia coli* has been extensively studied as the host bacterium, but the range and efficiency of the biosynthesis applications begin to be limited due to its intrinsic capabilities. Interestingly, *Vibrio* strains are gaining more and more attentions due to their rapid growth rates. For example, the doubling time of *Vibrio alginolyticus* and *Vibrio parahaemolyticus* is reported to be 12-14 min (Hoff et al., 2020; Li et al., 2021). Another bacterium *Vibrio natriegens* is reported to possess the fastest cell growth rate of known bacteria, with a doubling time of less than 10 min (Weinstock et al., 2016). Besides, the genomic editing tools of *Vibrio* have also been gradually established (Roh et al., 2012; Dalia et al., 2017; Lee et al., 2019). Therefore, the metabolic engineering of *Vibrio* strains as host bacteria for biological production would provide a new direction for the development of synthetic biology.

Polyhydroxyalkanoate (PHA) is an environment-friendly alternative to replace petroleum-based plastics due to its biodegradability, and the most common of which is poly-3-hydroxybutyrate (PHB) (Zheng et al., 2020). Generally, PHA is accumulated as intracellular lipid inclusion bodies to store carbon and energy in microbial cells (Choi et al., 2020; Pagliano et al., 2021). Up to now, there are relatively few studies on the PHA production using *Vibrio* strains. A few research indicated that *Vibrio* strain are responsible for PHA production in mixed microbial cultures (Cui et al., 2016; Zhao et al., 2021). Recently, *V. alginolyticus* and *V. proteolyticus* were reported as novel candidates for PHA production (Hong et al., 2019; Li et al., 2021). Studies have shown that the biosynthesis of PHA is a complex metabolic process, which involved a variety of enzymes (Meng et al., 2014). The most common pathway can be called the “three-step synthesis” pathway, in which the carbon source is converted into acetyl-CoA by microbial decomposition, and then two acetyl-CoA molecules are condensed into acetoacetyl-CoA by acetyl-CoA acetyltransferase (PhaA). Subsequently, 3-hydroxybutyryl-CoA is generated through the reduction of acetoacetyl-CoA under the catalysis of NADPH-dependent reductase (PhaB). Finally, PHA synthase (PhaC) polymerize the 3-hydroxybutyryl-CoA monomer to generate PHB (Sagong et al., 2018). There are also some other enzymes that can regulate PHA biosynthesis, such as the phasin family protein (PhaP) (Lee et al., 2021).

High production cost is a great limitation for the application of PHA as biodegradable materials (Liu et al., 2021). Industrial and agricultural wastes, such as volatile fatty acids (Pu et al., 2020a; Wang et al., 2022), waste potato starch (Haas et al., 2008), wheat straw (Dahman and Ugwu, 2014), and waste frying oil (Tian et al., 2022) have been proposed as inexpensive substrates to reduce the PHA cost. Starch is a biopolymer composed of glucose molecules, and regarded as one of the most abundant carbohydrates in nature. In terms of the molecular structure, starch is a macromolecular compound in which α-D-glucoside is connected by α-1,4-glycosidic linkages and α-1,6-glycosidic linkages (Hu et al., 2020). More than 70% of starch is used for the production of glucose, and amylase plays an important role in the process (Kmr et al., 2019). Amylase is a general term for enzymes with the ability to hydrolyze starch and glycogen, which could hydrolyze starch into low molecular structures (Chi et al., 2001; Zareian et al., 2010). Amylases are classified as α-amylase (EC 3.2.1.1) and β-amylase (EC 3.2.1.2) according to the type of sugar end groups generated during starch hydrolysis. The α-amylase, also known as α-1,4-glucose hydrolase, is more widely studied, with the ability of hydrolyzing starch into low molecular structures such as dextrin, maltose, and glucose (Chi et al., 2001). At present, many microorganisms have been employed to produce α-amylase, such as *Geobacillus* sp. (Mollania et al., 2010), *Bacillus subtilis* (Asgher et al., 2007), *B. licheniformis* (Shewale and Pandit, 2007), *B. stearothermophilu*s (Chakraborty et al., 2000), and *Aspergillus oryzae* (Henriksen et al., 1999).

Previously, *V. alginolyticus* LHF01 isolated from a salt field was found to produce PHB from a variety of sugars and organic acids (Li et al., 2021). In this study, a genetic manipulation platform was developed for *V. alginolyticus* LHF01, and based on this, the utilization of starch by the strain and the overexpression of PHB synthesis operon and amylase were investigated. The direct utilization of starch by *V. alginolyticus* LHF01 would be helpful to reduce the cost of PHA production, making it as a promising bacterium for PHA production to achieve a breakthrough achievement in PHA commercialization.

## 2 Materials and methods

### 2.1 Strains and plasmids

Bacterial strains and plasmids used in this study are listed in Table 1. *E. coli* strain JM109 was used for molecular cloning and plasmid propagation, while *E. coli* strain S17-1 was used as the doner strain for conjugation (Simon et al., 1983). *V. alginolyticus* LHF01 with their engineered strains were employed for PHB production experiments.

**TABLE 1:** Strains and plasmids used in this study.

Name	Relevant characteristics	Reference
Strains		
*E. coli* JM109	*recA1 endA1 gyrA96 thi*-1 *hsdR17 supE44 relA1 Δ*(*lac*-*proAB*)/F’ [*traD36 proAB* ^+^ *lacI* ^q^ *lacZΔ*M15]	TaKaRa Bio Inc
*E. coli* S17-1	Donor strain in conjugation, harboring the *tra* genes of plasmid RP4 in the chromosome	Simon et al. (1983)
*V. alginolyticus* LHF01	Wild type, isolated from a salt field in China	This study
LHF01 (phaBAPC)	*V. alginolyticus* LHF01 harboring pMCS1-phaBAPC	This study
LHF01 (03151)	*V. alginolyticus* LHF01 harboring pMCS1-03151	This study
LHF01 (03416)	*V. alginolyticus* LHF01 harboring pMCS1-03416	This study
LHF01 (03713)	*V. alginolyticus* LHF01 harboring pMCS1-03713	This study
LHF01 (03713-phaBAPC)	*V. alginolyticus* LHF01 harboring pMCS1-03713-phaBAPC	This study
Plasmids		
pBBR1MCS-2	Broad range host plasmid, Kan^R^	Kovach et al. (1995)
pSEVA341	Expression vector, Cat^R^	Tan et al. (2022)
pMCS1	Broad range host plasmid, Cat^R^	This study
pMCS1-phaBAPC	pMCS1 derived, carrying *phaBAPC* of LHF01	This study
pMCS1-03151	pMCS1 derived, carrying amylase gene 03151 of LHF01	This study
pMCS1-03416	pMCS1 derived, carrying amylase gene 03416 of LHF01	This study
pMCS1-03713	pMCS1 derived, carrying amylase gene 03713 of LHF01	This study
pMCS1-03713-phaBAPC	pMCS1 derived, carrying amylase gene 03713 and *phaBAPC* of LHF01	This study

The primers used in this study are listed in Supplementary Table S1. To overexpress the target genes in *V. alginolyticus* LHF01, pMCS1 was constructed by ligating the plasmid backbone fragment amplified from pBBR1MCS-2 (Kovach et al., 1995) with primers P1_F/R and the chloramphenicol fragment amplified from pSEVA341 (Tan et al., 2022) with primers cat_F/R by Gibson assembly. Plasmid pMCS1-phaBAPC was constructed to overexpress the PHB synthesis operon *phaBAPC* of *V. alginolyticus* LHF01. The *phaBAPC* and its native promoter were amplified from the genome of *V. alginolyticus* LHF01 using primers phaBAPC_F/R, and then inserted into the *Eco*RI/*Bam*HI sites of pMCS1 to generate pMCS1-phaBAPC. Similarly, pMCS1-03151, pMCS1-03416, and pMCS1-03713 were constructed to overexpress the amylase of *V. alginolyticus* LHF01. Primers amy2_F/R, amy3_F/R, and amy4_F/R were used to amplify the three different amylase gene and their own promoter, respectively. Then, the DNA fragment was inserted into the multiple cloning sites of pMCS1 by enzyme digestion and ligation. Plasmid pMCS1-03713-phaBAPC was constructed to co-overexpress *phaBAPC* and amylase gene 03713. The amylase gene fragment amplified from *V. alginolyticus* LHF01 using primers amy4_phaBAPC_F/R were digested with *Eco*RI/*Kpn*I and then cloned into the corresponding sites of pMCS1-phaBAPC to construct pMCS1-03713-phaBAPC.

### 2.2 Medium and culture conditions

During all experiments, *E. coli* was cultured in Luria-Bertani (LB) medium, while *V. alginolyticus* was cultured in TYS50 medium. TYS50 medium contained (g/L) NaCl 50, KCl 0.7, CaCl_2_·2H_2_O 1.4, MgSO_4_·7H_2_O 6.8, MgCl_2_·6H_2_O 5.4, NaHCO_3_ 0.2, yeast extract 1, and peptone 5. When required, 1.5% (w/v) agar was added to obtain the corresponding solid medium. In the process of culturing *V. alginolyticus* LHF01 for PHB production, soluble starch was firstly used as carbon source, and the shake flask experiments were performed at 37°C and 200 rpm. The culture conditions were optimized in terms of pH, soluble starch concentration, and culture time. The pH was set at 5, 6, 6.5, 7, 7.5, 8, 8.5, 9, and 10, while the starch concentration gradient was 20, 30, and 40 g/L. The samples were taken every 2 hours to determine the cell growth, PHB accumulation, and starch consumption.

### 2.3 Genome sequencing

The genomic DNA of *V. alginolyticus* LHF01 was extracted using a Blood & Cell Culture DNA Midi Kit (Qiagen, United States) according to the manufacturer’s protocol. DNA concentration and purity were determined *via* Qubit fluorometer and Nanodrop 2000 spectrophotometer (Thermo Fisher Scientific, United States). DNA integrity was assessed by 1% agarose gel electrophoresis. Whole genome sequencing was performed on the MGISEQ-2000 platform and PacBio Sequel II system at BGI (Shenzhen, China).

### 2.4 Conjugation methods

The target plasmid was transferred into *V. alginolyticus* LHF01 by conjugation. In brief, the plasmid was firstly transformed into *E. coli* S17-1 to prepare the donor strain. *E. coli* S17-1 and *V. alginolyticus* strains were cultured in LB and TYS50 medium, respectively, with the relevant antibiotics to an OD_600_ of 1.5-2. Afterwards, 1.5 mL of donor and recipient cells were respectively placed in a centrifuge tube and centrifuged at 6,000 rpm for 5 min. The supernatant was discarded, and the cell pellets were washed once with 1 mL of the corresponding medium, and then suspended in 50 μL of TYS50 medium at a ratio of 1:1. The suspension was cultured for 4-8 h in a 37°C incubator. Finally, an inoculation loop was dipped into the bacterial solution, and then streaked on the TYS50 medium plate with appropriate antibiotics. The plate was cultured in a 37°C incubator for 36-48 h. A single clone was picked out, and put into the TYS50 medium with antibiotics for 24-48 h, and the bacterial solution was verified by 16S rDNA sequencing and stored for follow-up research.

### 2.5 Starch consumption and PHA production analysis

After treating with boiling water, the soluble starch can be dissolved and its solution is transparent at room temperature. Since starch is turned to blue when exposed with iodine, its consumption could be indicated by adding the iodine solution. To measure PHB accumulation, the strain culture was centrifuged at 10,000 g for 10 min, and washed twice with deionized water. Then the cell pellet was lyophilized to determine the cell dry weight (CDW) and PHB content. CDW was calculated based on the weight of the empty centrifuge tube, the weight of the centrifuge tube containing the cell pellet, and the volume of freeze-dried culture. The lyophilized cell was placed into an esterification tube, reacted with 2 mL of chloroform and 2 mL of esterification solution at 100°C for 4 h, and then shaken with 1 mL of deionized water, and set for stratification. The chloroform phase was taken for gas chromatography (GC) analysis as reported previously (Li et al., 2021).

### 2.6 Observation of cell morphology

In order to observe the cell morphological changes of *V. alginolyticus* strains when overexpressing the PHB synthesis operon, the wild type LHF01 and engineered recombinants were cultured in TYS50 medium with 20 g/L soluble starch for 18 h and observed by transmission electron microscopy (TEM). The shake flask culture was centrifuged to obtain the cell pellet, washed twice with pre-cooled phosphate buffered solution, slowly added with pre-cooled fixative solution, and then fixed for 12 h to obtain the cell samples. Then, the sample was sliced and observed according to the methods reported previously (Li et al., 2021).

## 3 Results and discussion

### 3.1 PHB production by *V. alginolyticus* using soluble starch

PHA is regarded as extremely competitive alternative to replace the petrochemical plastics, yet its high cost is always a major impediment. There have been numerous researches working on the exploitation of low-cost substrates and novel production hosts (Anjali et al., 2014; Vibhavee and Chanprateep, 2018). As an industrial raw material, starch has been applied for the production of high value-added products such as gas or liquid fuels, proteins, and sugars (Erdei et al., 2010; Lin et al., 2021; Li et al., 2022). However, many microorganisms are unable to utilize starch directly, and the cost of raw materials is increased due to the pretreatment of starch (Poomipuk et al., 2014). Interestingly, *Vibrio* strains have attracted increasing attentions due to their rapid growth rate and ability to assimilate starch (Hoff et al., 2020). *V. alginolyticus* was reported to accumulate PHB from a series of carbon sources (Li et al., 2021). The direct utilization of starch for PHA production and metabolic engineering were explored in *V. alginolyticus* LHF01 in this study.

**FIGURE 1:**
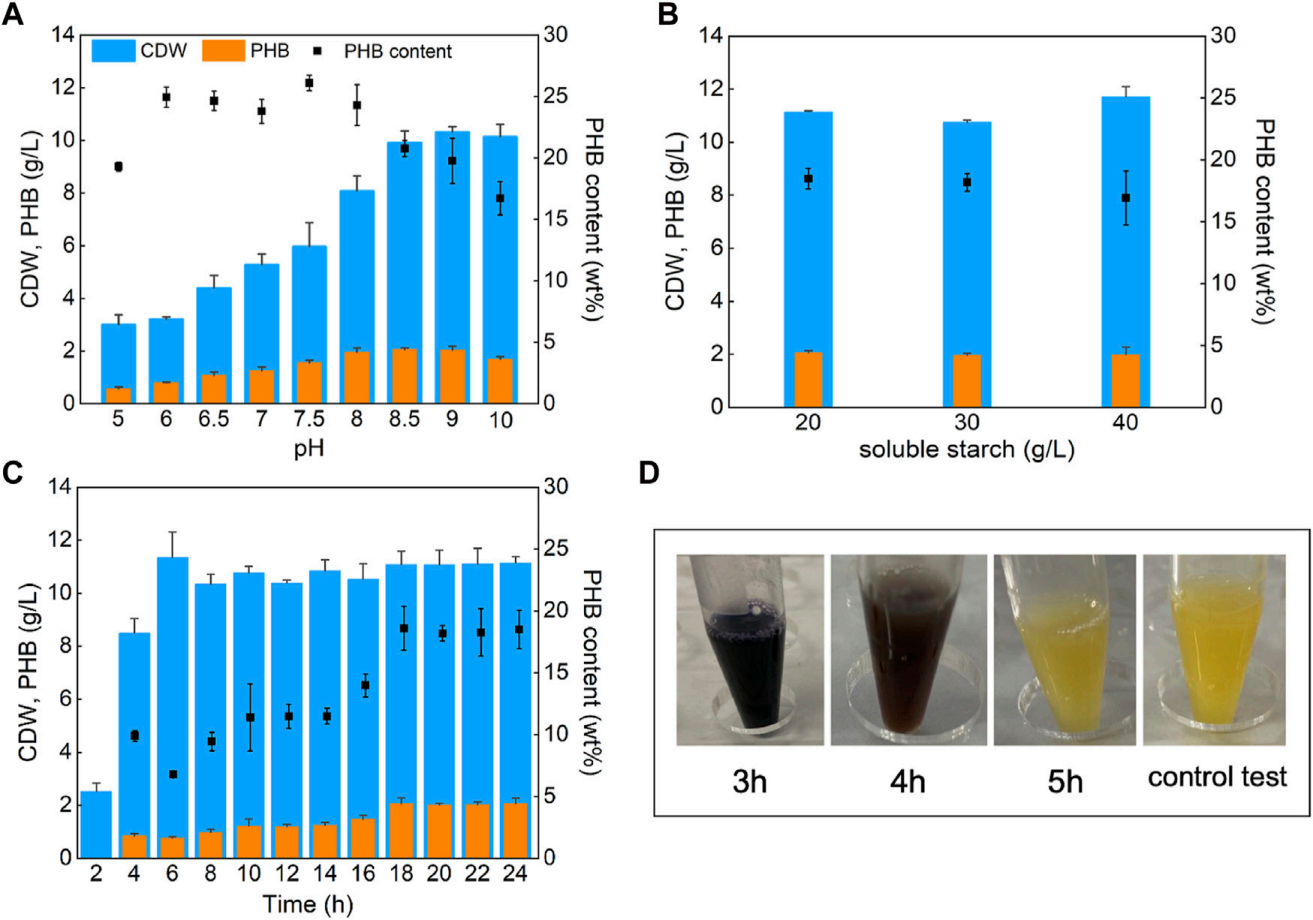
Effects of pH, substrate concentration, and culture time on PHB production. *V. alginolyticus* LHF01 was cultivated in TYS50 medium in shake flasks at 37°C, 200 rpm. Data are expressed as averages and standard deviations of three parallel experiments. **(A)**, the pH was set at 5, 6, 6.5, 7, 7.5, 8, 8.5, 9, and 10. **(B)**, the starch concentration gradient was 20, 30, and 40 g/L. **(C)**, the samples were taken every 2 hours to determine CDW and PHB accumulation. **(D)**, starch consumption was indicated by adding iodine solution to the cultures.

*V. alginolyticus* LHF01 was firstly cultured with 20 g/L soluble starch as carbon source for 24 h to explore the effect of pH on PHB production (Figure 1A). Although the difference in pH had a certain effect on the CDW and PHB titers, *V. alginolyticus* LHF01 can synthesize PHB under a wide range of pH, indicating that it has strong viability and adaptable ability. PHB production by *V. alginolyticus* LHF01 showed an upward trend with the increase of pH value. When the pH was 8-10, CDW exceeded 8 g/L, and PHB titer was about 2 g/L, indicating that *V. alginolyticus* LHF01 is more suitable for survival under alkaline conditions. The highest titer PHB of 2.06 g/L was obtained at pH of 8.5. Therefore, the pH of the culture medium was maintained around 8.5 in the subsequent experiments of culturing *V. alginolyticus* LHF01.

Since the change of substrate concentration may also affect the growth of bacteria, the concentration of initial soluble starch was increased to 40 g/L to observe the change in PHB production. As shown in Figure 1B, the CDW and PHB titer did not change significantly with the increased soluble starch concentration. A possible reason was that the strain reached the growth threshold at 20 g/L of soluble starch, thus the increase of carbon concentration showed no assistance to further promote PHB accumulation. Then, *V. alginolyticus* LHF01 was cultured with a soluble starch concentration of 20 g/L for different time points to study the cell growth and PHB accumulation profiles. As shown in Figure 1C, the CDW of *V. alginolyticus* LHF01 reached 2.52 g/L after cultured for 2 h, up to 8.49 g/L for 4 h, and exceeded 10 g/L after 6 h, which confirmed the rapid cell growth of *V. alginolyticus* in shake flasks. Surprisingly, the PHB titer of *V. alginolyticus* LHF01 was only 0.84 g/L at 4 h of cultivation, indicating that PHB was synthesized during the late stage of cell growth. After 18 h of cultivation, PHB titer reached the highest level. It is well known that starch or soluble starch is turned to blue when exposed to iodine. To explore the consumption rate of soluble starch, iodine was used to determine whether soluble starch was completely consumed. As shown in Figure 1D, soluble starch was exhausted by *V. alginolyticus* LHF01 after 5 h of cultivation, indicating that the strain has superior ability to degrade soluble starch. The rapid cell growth and efficient utilization of starch make *V. alginolyticus* a promising candidate for microbial production of building block chemicals or polymers using starch as the substrate.

### 3.2 Complete genome sequencing of *V. alginolyticus* LHF01

**FIGURE 2:**
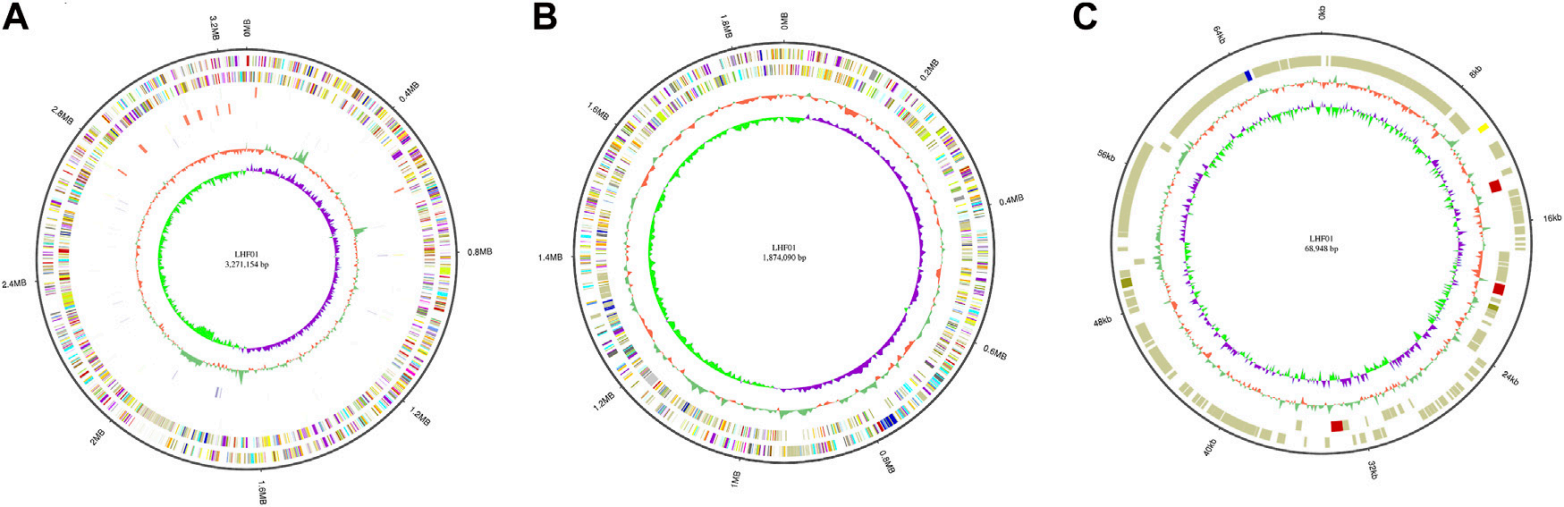
Genome map of *V. alginolyticus* LHF01. **(A)**, chromosome 1. **(B)**, chromosome 2. **(C)**, endogenous plasmid.

Whole genome sequencing has the highest resolution in bacterial genetic research, and is widely used in microbial traceability, transmission, and population structure identification. Therefore, the genomic characterization of *V. alginolyticus* LHF01 was performed. Its complete genome was sequenced and reassembled from Celera Assembler 8.3 (Denisov et al., 2013). The complete genome sequence was deposited in GenBank database under the accession numbers of CP087876.1, CP087877.1, and CP087878.1. Results indicated that the genome of *V. alginolyticus* LHF01 contains two chromosomes and one plasmid (Figure 2). The full length of chromosome one is 3,271,154 bp, with GC content of 44.74%, and the full length of chromosome two is 1,874,090 bp, with GC content of 44.68%. The full length of plasmid DNA was 68,948 bp and GC content was 42.24%. The annotation was carried out with the National Centre for Biotechnology Information Prokaryotic Genomes Automatic Annotation Pipeline (PGAAP) Version 4.9 (https://www.ncbi.nlm.nih.gov/genome/annotation_prok). From the 4,882 predicted genes identified, a total of 4,639 protein-coding sequences (CDSs) were determined, and 128 tRNA genes, 37 complete rRNA genes (13 for 5S, and 12 each for 16S and 23S), and four ncRNAs were annotated.

In the metabolic pathways of starch degradation and PHB production, amylase and PHB synthesis-related enzymes are indispensable. There are multiple genes encoding amylase, and the gene locus tag number is 03151, 03416, and 03713, respectively. A phylogenetic tree of the amylases of *V. alginolyticus* LHF01 was constructed, and results indicated that they are all α-amylase and belong to the glycoside hydrolase family 13 (Kuriki et al., 2006) (Supplementary Figure S1).

Previous studies reported that there were genes encoding PHA synthesis enzymes existed in the genome of *Vibrio* strains including *V. harvinii* (Mohandas et al., 2016), *V. natriureus* (Chien et al., 2007), and *V. azureus* (Sasidharan et al., 2014). Genome analysis revealed the presence of PHB synthesis operon *phaBAPC* in *V. alginolyticus* LHF01. The operon comprised of four genes including *phaB*, *phaA*, *phaP*, and *phaC*, encoding acetoacetyl-CoA reductase, acetyl-CoA acetyltransferase, phasin, and PHA synthase, respectively. The amino acid sequence of PHA synthase in *V. alginolyticus* LHF01 belongs to Class I PHA synthase. The genes responsible for PHA biosynthesis usually clustered together in the bacterial genome (Rehm and Steinbüchel, 1999), such as the most well-known genetic organization of *phaCAB* operon in *Ralstonia eutropha* H16 (Peoples and Sinskey, 1989), the *phaCA* operon in *Jeongeupia* sp. USM3 (Zain et al., 2020), the *phaEC* operon in *Neptunomonas concharum* (Pu et al., 2020b). Interestingly, the presence of phasin gene in a PHA operon is rarely reported. The neighborhood genetic organization of *phaP* and *phaA*, *phaB*, *phaC* genes may play a unique role in the regulation of PHB metabolism in *V. alginolyticus*, which deserves further study. The genome sequencing of *V. alginolyticus* would provide a good foundation for further in-depth metabolic engineering for improved substrate utilization and PHB production.

### 3.3 Effects of overexpressing PHB synthesis operon on PHB production

**FIGURE 3:**
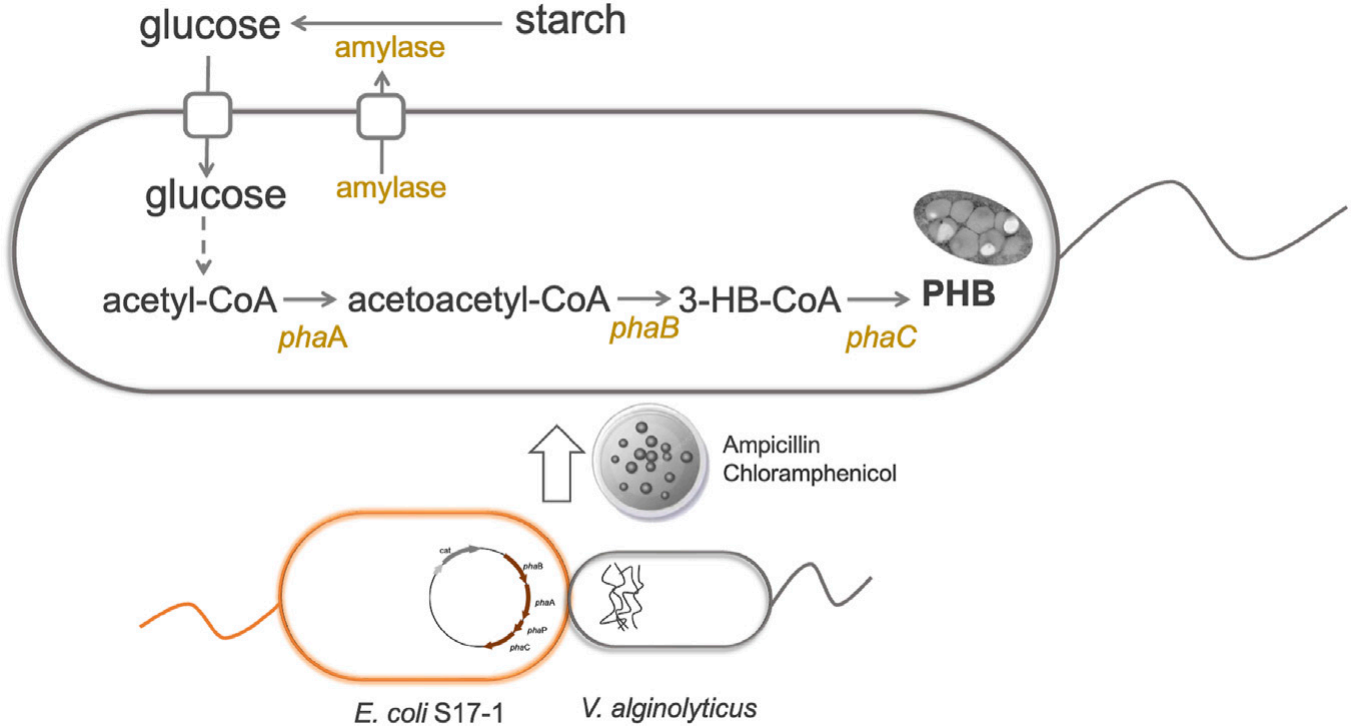
Construction of a genetic manipulation platform for improved PHB production in *V. alginolyticus* LHF01.

The genetic modification of host cells is one of the most powerful tools in engineering bacteria for microbial fermentation. To begin with, molecular biology research requires introducing foreign plasmids into the target hosts. Generally, developing DNA transformation methods for non-model organism poses unknown challenges. Fortunately, the broad host pBBR1 origin was reported to be transformable into *V. natriegens via* conjugation (Tschirhart et al., 2019). No chloramphenicol resistance was observed for *V. alginolyticus* (Li et al., 2021), thus plasmid pMCS1 harboring pBBR1 origin and chloramphenicol resistance gene was constructed to study its possible application to transfer foreign DNA into *V. alginolyticus* LHF01. We firstly tried electroporation and chemical transformation using pMCS1 as the shuttle vector. However, it was found that both methods failed and no transformant was observed despite of many attempts. Recently, the deletion of gene clusters involved in the biosynthesis of exopolysaccharides and O-antigen was proved to improve the permeability of exogenous DNA into cells and enabled the electrotransformation in *Halomonas bluephagenesis* (Xu et al., 2022). The electroporation method for *V. alginolyticus* LHF01 was worthy of further investigation. Next, a DNA transformation procedure through conjugation was established and pMCS1 was successfully introduced into *V. alginolyticus* LHF01 by using *E. coli* S17-1 as the assistant strain (Figure 3). Overexpression of the endogenous PHA synthesis genes is a common metabolic strategy for directing the carbon metabolic flux towards PHA synthesis pathway and strengthening PHA production (Khetkorn et al., 2016; Zhao et al., 2019; Tang et al., 2022). For example, the application of promoter engineering to enhance transcription of PHA synthase gene increased PHA accumulation in *Pseudomonas mendocina* (Zhao et al., 2019). To explore the role of native PHB synthesis genes on the production of PHB, *phaBAPC* operon was cloned with the native promoter and ligated into pMCS1 to construct the plasmid pMCS1-phaBAPC. The constructed plasmid was transferred into *V. alginolyticus* LHF01 by conjugation to obtain the recombinants with ability to overexpress the PHB synthesis operon. The recombinant strain LHF01 (phaBAPC) was verified by 16S rDNA sequencing.

**FIGURE 4:**
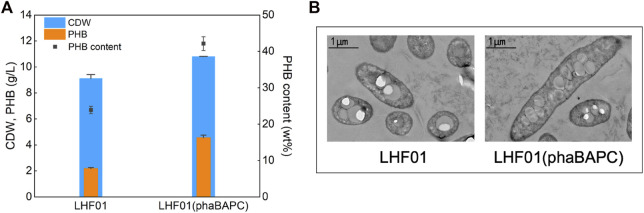
Effects of overexpressing PHB synthesis operon on PHB production.Strains were cultivated in TYS50 medium supplemented with 20 g/L soluble starch in shake flasks at 37°C and 200 rpm for 18 h **(A)**, CDW, PHB production, and PHB content in LHF01 and LHF01 (phaBAPC). **(B)**, TEM micrographs of LHF01 and LHF01 (phaBAPC).

Soluble starch was used to cultivate the recombinant strain harboring extra *phaBAPC* operon and the wild type control. As shown in Figure 4A, PHB titer of 4.55 g/L and PHB content was 42.13% were obtained in strain LHF01 (phaBAPC). The PHB titer was increased by more than 100% compared with the control strain LHF01. Therefore, the overexpression of *phaBAPC* can well promote the conversion of soluble starch to PHB, and it also demonstrates the success of genetic manipulation in *V. alginolyticus*. In addition, the cells of *V. alginolyticus* LHF01 and LHF01 (phaBAPC) grown on soluble starch were observed by TEM. It was obvious that electron translucent inclusions can be observed in both bacterial cells, yet LHF01 (phaBAPC) has significantly more intracellular particles than *V. alginolyticus* LHF01 (Figure 4B). Both PHA synthase and phasins existed on the surface of PHA granules by covalent and hydrophobic interactions (Cai et al., 2011). It was proposed that the phasins affected the activity of PHA synthase and determined the number and size of PHA granules (Mezzina and Pettinari, 2016). Herein, similar phenomenon was also observed in *V. alginolyticus*, and the overexpression of *phaBAPC* is beneficial to the PHB production.

### 3.4 Effects of overexpressing amylase on PHB production

**FIGURE 5:**
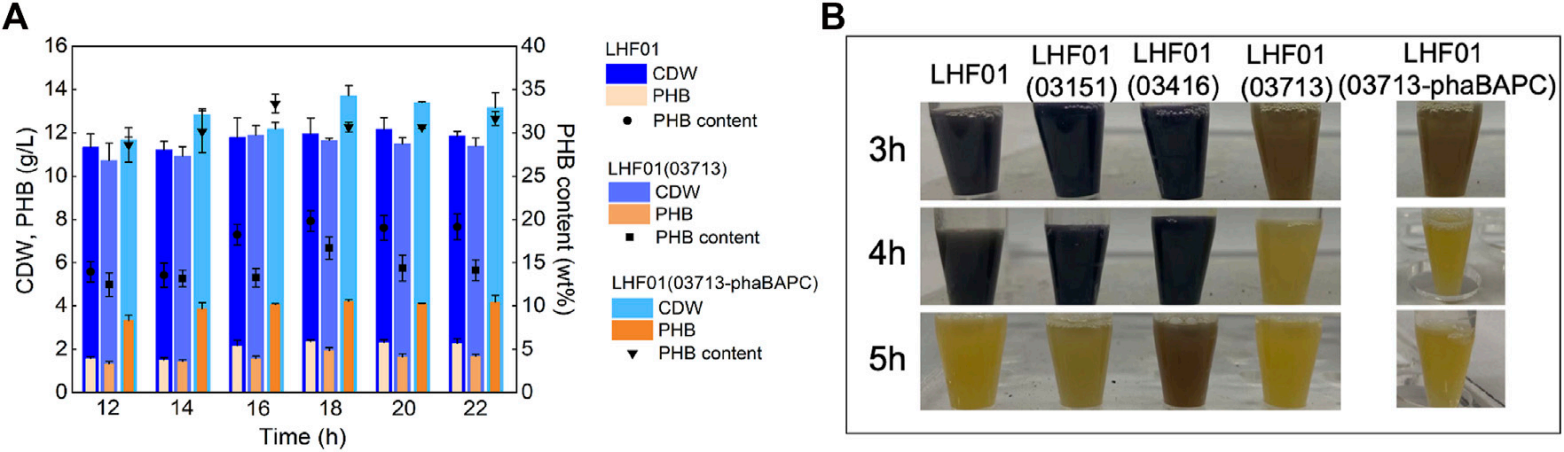
Effects of co-overexpressing amylase and PHB synthesis operon on PHB production.Strains were cultivated in TYS50 medium supplemented with 20 g/L soluble starch in shake flasks at 37°C and 200 rpm. **(A)**, CDW, PHB production, and PHB content in LHF01, LHF01 (03713), and LHF01 (03713-phaBAPC). **(B)**, starch consumption was indicated by adding iodine solution to the cultures.

Next, the three amylase genes of *V. alginolyticus* LHF01 were cloned to construct plasmids pMCS1-03151, pMCS1-03416, and pMCS1-03713, respectively. Recombinants LHF01 (03151), LHF01 (03416), and LHF01 (03713) were obtained by conjugation. Shake flask experiments were performed to study the consumption rate of soluble starch. As shown in Figure 5B, the samples of LHF01 (03151), LHF01 (03416), and control strain LHF01 were no longer turned blue at about 5 h, while the sample of LHF01 (03713) was turned yellow at 3-4 h. Among the three amylases, the overexpression of amylase 03713 significantly increased the consumption of soluble starch. However, the overexpression of amylase did not effectively improve PHB accumulation (Supplementary Figure S2).

Afterwards, strain LHF01 (03713-phaBAPC) was constructed to explore the synergistic effect of PHB synthesis operon and amylase on substrate consumption and PHB accumulation. The PHB titer of *V. alginolyticus* LHF01, LHF01 (03713) and LHF01 (03713-phaBAPC) all reached the maximum at 18 h, but it was obvious that LHF01 (03713-phaBAPC) had the PHB highest titer of 4.20 g/L at this time, and the PHB content reached 30.66% (Figure 5A). Furthermore, the culture of LHF01 (03713-phaBAPC) was turned to yellow at 3-4 h, indicating that LHF01 (03713-phaBAPC) exhibited faster consumption rate of soluble starch compared with *V. alginolyticus* LHF01 (Figure 5B). It can be said that these phenomena indicated that the co-expression of 03713 gene with *phaBAPC* not only increased the rate of soluble starch consumption, but also increased the titer of PHB.

**FIGURE 6:**
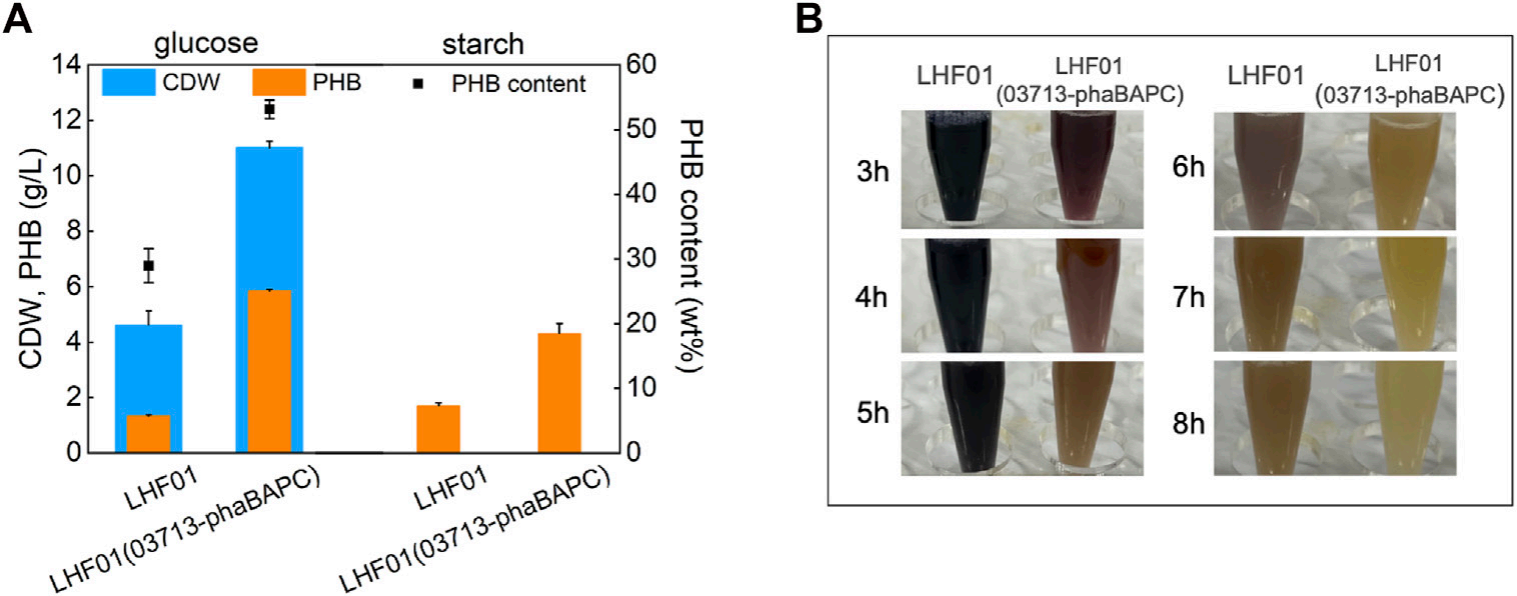
PHB production by *V. alginolyticus* strains using corn starch or glucose.Strains were cultivated in TYS50 medium supplemented with 20 g/L insoluble corn starch or glucose in shake flasks at 37°C and 200 rpm. **(A)**, CDW, PHB production, and PHB content in LHF01 and LHF01 (03713-phaBAPC). **(B)**, starch consumption was indicated by adding iodine solution to the cultures.

It had to be admitted that the cost of soluble starch is relatively high due to the reliance on chemical or other means to denature starch. Therefore, soluble starch was replaced with insoluble corn starch to explore the direct utilization of starch by *V. alginolyticus.* Strain LHF01 and LHF01 (03713-phaBAPC) were cultivated in TYS50 medium supplemented with 20 g/L corn starch for 18 h, and starch consumption and PHB titer were recorded. Glucose was also employed as the control. Due to the insolubility of corn starch, the accurate CDW cannot be measured. The PHB titer of wide type strain LHF01 was 1.70 g/L, a litter higher than that of 1.33 g/L when glucose was used as the carbon source. In terms of corn starch consumption, starch was not completely consumed after 8 h cultivation by LHF01, showing that the utilization ability of corn starch was weaker than that of soluble starch. It is worth mentioning that, the PHB titer of LHF01 (03713-phaBAPC) reached 4.32 g/L with corn starch, slightly lower than that of 5.85 g/L using glucose as carbon source (Figure 6A). In addition, the sample of LHF01 (03713-phaBAPC) was turned into yellow at 6-7 h, indicating that the starch consumption ability of LHF01 (03713-phaBAPC) was much higher than the control strain LHF01 (Figure 6B).

Several strains have been reported to utilize starch for PHA production, including *Aeromonas*, *Bacillus cereus*, *Massilia*, *Halogeometricum borinquense*, and *Halolamina* (Table 2). Generally, the PHA titers and PHA content in the isolated wild type strains were not high. To improve starch utilization and PHA accumulation, metabolic engineering strategies have been applied in *E. coli* and *H. bluephagenesis*, which cannot assimilate starch naturally. The recombinant *E. coli* carrying amylase gene from *Panibacillus* sp. and PHB synthesis genes from *R. eutropha* accumulated 1.24 g/L of PHB using starch (Bhatia et al., 2015). Engineered *H. bluephagenesis* harboring an amylase with suitable signal peptide was able to grow on corn starch to 9.5 g/L cell dry weight containing 51.5% PHB (Lin et al., 2021). Compared to previous reported studies, *V. alginolyticus* LHF01 indicated the fastest starch consumption rate. For example, the engineered *V. alginolyticus* could exhaust 20 g/L corn starch in 7 h and accumulate 4.32 g/L PHB after 18 h cultivation, while the shake flask experiments for PHA production by other reported strains using starch were usually performed for 48 h or even longer. These results demonstrated *V. alginolyticus* to be a superior chassis for production of PHB from low-cost corn starch. Further development of genetic manipulation techniques and rational engineering will help to improve PHB production from starch by *V. alginolyticus*.”

**TABLE 2:** PHA production by microorganisms using starch as the carbon source.

Strain	Carbon source	Products	CDW (g/L)	PHA content (wt%)	PHA (g/L)	Reference
*Aeromonas* sp. KC007-R1	Starch	PHB	1.83	32.7	0.6	Chien and Ho (2008)
*Bacillus cereus* CFR06	Soluble starch	PHB	2.14	46	1.0	Halami (2008)
*Massilia* sp. UMI-21	Corn starch	PHB	3.95	30.3	1.20	Han et al. (2014)
*Halogeometricum borinquense*	Soluble starch	PHBV	6.2	74.19	4.6	Salgaonkar et al. (2019)
*Halorubrum chaoviator* CEJ34-14	Soluble starch	PHB	0.22	9.25	0.02	Karray et al. (2021)
*Natrinema pallidum* CEJ5-14	Soluble starch	PHB	0.56	7.11	0.04	Karray et al. (2021)
*Halolamina* sp. NRS_35	Starch	PHB	-	-	0.042	Hagagy et al. (2022)
*Halolamina* sp. NRS_38	Starch	PHB	-	-	0.037	Hagagy et al. (2022)
*E. coli* SKB99	Starch	PHB	2.16	57.4	1.24	Bhatia et al. (2015)
*Halomonas bluephagenesis* TN04	Corn starch	PHB	9.5	51.5	4.89	Lin et al. (2021)
*V. alginolyticus*	Soluble starch	PHB	10.80	42.13	4.55	This study
*V. alginolyticus*	Corn starch	PHB	-	-	4.32	This study

## 4 Conclusion

In this study, a genetic manipulation platform based on conjugation was established for the first-time in *V. alginolyticus* LHF01, and based on this, the utilization of starch by the strain and the overexpression of PHB synthesis operon and amylase were investigated. *V. alginolyticus* LHF01 was able to consume 20 g/L soluble starch in 5 h shake flask cultivation, achieving PHB titer of 2.06 g/L. Afterwards, the genome of *V. alginolyticus* LHF01 was sequenced and analyzed. With the overexpression of its *phaBAPC* operon, PHB titer was improved to 4.55 g/L, and the consumption of soluble starch was increased with the overexpression of its native amylase. Notably, the engineered *V. alginolyticus* harboring extra amylase and PHB synthesis operon was successfully used to degrade 20 g/L corn starch in 7 h shake flask cultivation, with PHB titer of 4.32 g/L. The use of *V. alginolyticus* points to the possibility of rapid cell growth for production of chemicals and biopolymers using starch, a sustainable resource with cost much lower than glucose. Further engineering of *V. alginolyticus* may open new applications in metabolic engineering and synthetic biology research.

## Data Availability

The datasets presented in this study can be found in online repositories. The names of the repository/repositories and accession number(s) can be found below: https://www.ncbi.nlm.nih.gov/genbank/, CP087876.1. https://www.ncbi.nlm.nih.gov/genbank/, CP087877.1. https://www.ncbi.nlm.nih.gov/genbank/, CP087878.1.
