# Supplementary material for: Engineering Vibrio alginolyticus as a novel chassis for PHB production from starch
Source: Front Bioeng Biotechnol. 2023 Feb 7;11:1130368. doi: 10.3389/fbioe.2023.1130368 (PMC9941669; doi:10.3389/fbioe.2023.1130368)
Supplement: Supplementary file 1 [file DataSheet1.docx]

**Supporting information**

Fig. S1 Phylogenetic tree of the amylases in *V. alginolyticus* LHF01.


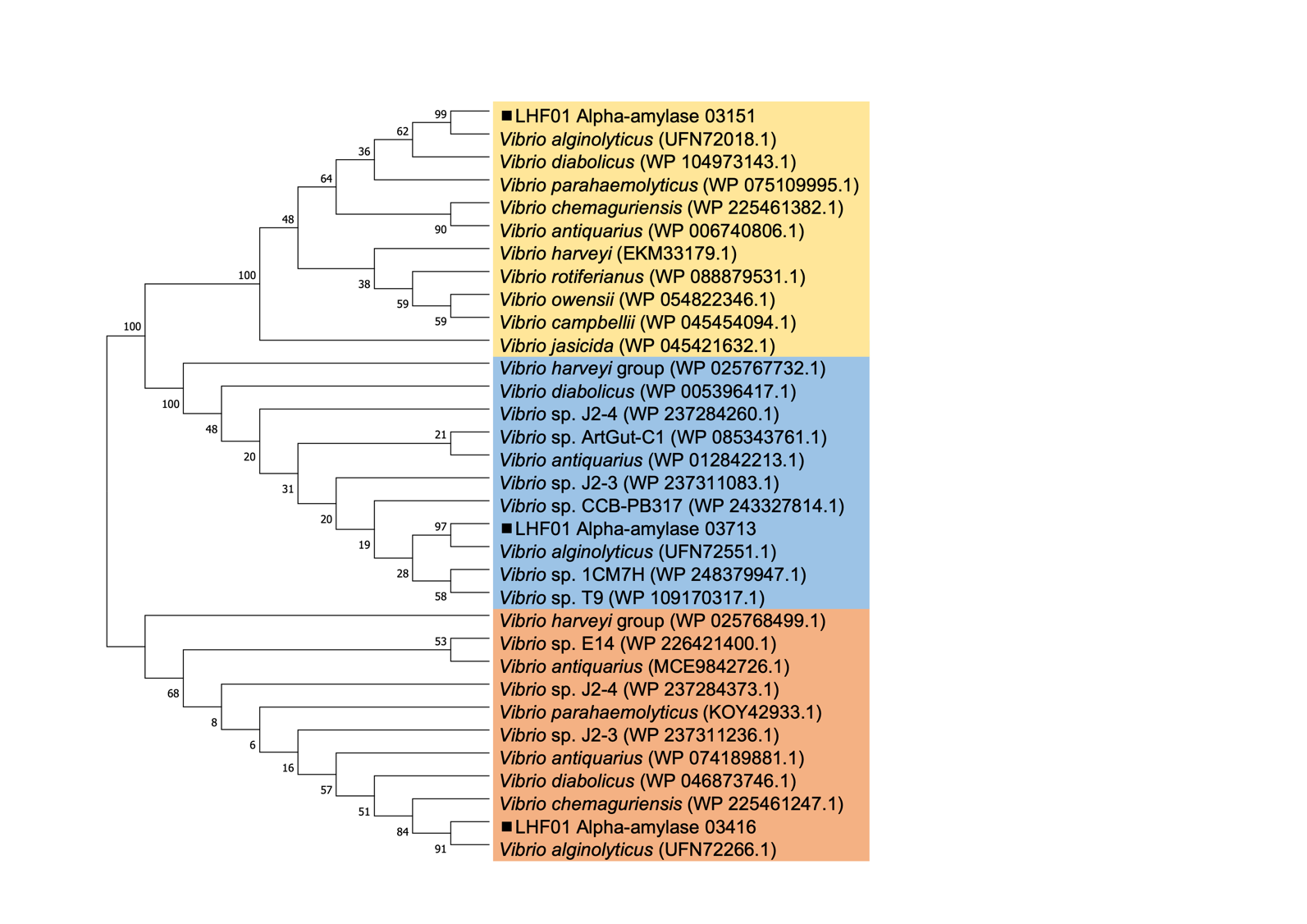


GenBank accession numbers were in the parentheses after bacterial names. Numbers at the branch ends are the bootstrap values based on 1000 replicates.


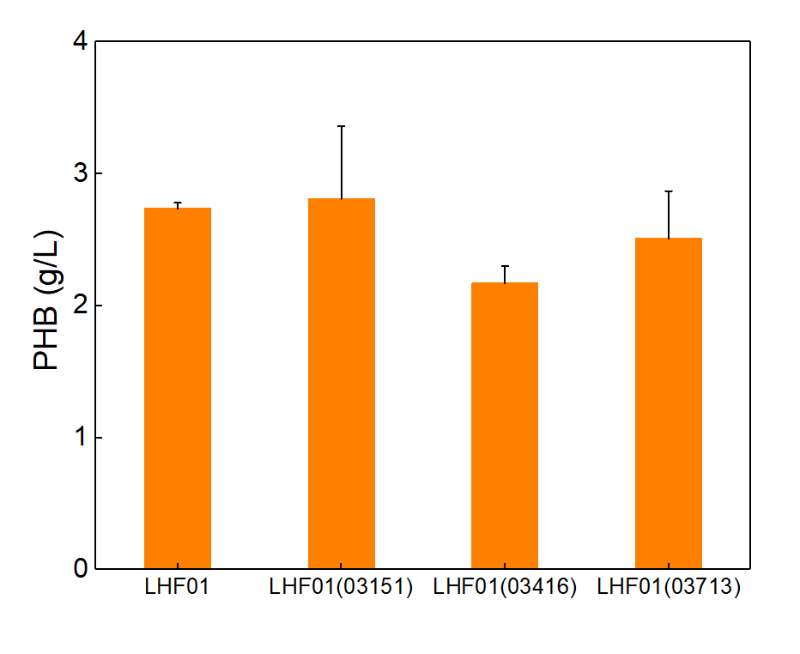
Fig. S2 Effects of amylase overexpression on PHB production in *V. alginolyticus*.

Strains were cultivated in TYS medium supplemented with 20 g/L soluble starch in shake flasks at 37 ℃ and 200 rpm for 18 h. Data are expressed as averages and standard deviations of three parallel experiments.

Table S1 Primers used in this study.

| Name | Primer sequences |
| --- | --- |
| P1_F | CGGGGCGTAACTGTCAGACCAAGTTTACTCGGTAC |
| P1_R | CGTGCCGATCACACATTTCCCCGAAAAGTGCAGA |
| cat_F | GGGAAATGTGTGATCGGCACGTAAGAGGTTCCA |
| cat_R | GGTCTGACAGTTACGCCCCGCCCTGC |
| phaBAPC_F | CCGGAATTCGCGAACTGCACCAAAATGAGACGTT |
| phaBAPC_R | CGCGGATCCCGGTATAATAAGGTAGTCAGTAACGA |
| amy2_F | CCGGAATTCCCCTCAATCCATTGCGCTTAA |
| amy2_R | CGCGGATCCAACTAAAACAACAAAGCCCTTCCT |
| amy3_F | CCGCTCGAGTTAAGGTTGGATACGCAGAAGGG |
| amy3_R | CGCGGATCCACATTAGACTTTACCCGTTCTTGA |
| amy4_F | CCGGAATTCTCTTAAATGACTGGCTTTCTTCGTT |
| amy4_R | CGCGGATCCATCAAATAACGTATTGATTCACTGCAA |
| amy4_phaBAPC_F | CTGTCAGACCAAGTTTACTCGGTACCTCTTAAATGACTGGCTTTCTTCGT |
| amy4_phaBAPC_R | ACGTCTCATTTTGGTGCAGTTCGCGAATTCATCAAATAACGTATTGATTCACTGCAAAG |
